# Beyond MVPA minutes: physical activity phenotypes are associated with metabolic profiles in children with obesity

**DOI:** 10.1007/s00431-026-07299-2

**Published:** 2026-08-06

**Authors:** Alessandro Gatti, Matteo Vandoni, Caterina Cavallo, Vittoria Carnevale Pellino Carnevale Pellino, Virginia Rossi, Anna Odone, Gianvincenzo Zuccotti, Valeria Calcaterra

**Affiliations:** 1 Laboratory of Adapted Motor Activity (LAMA), Department of Public Health, Experimental Medicine and Forensic Science, University of Pavia, 27100 Pavia, Italy; 2 National PhD Programme in One Health Approaches to Infectious Diseases and Life Science Research, Department of Public Health, Experimental and Forensic Medicine, University of Pavia, 27100 Pavia, Italy; 3 Luxembourg Health & Sport Sciences Research Institute A.S.B.L., 50, Avenue du Parc des Sports, Differdange, 4671 Luxembourg; 4 Department of Education and Sport Sciences, Pegaso University, 80143 Naples, Italy; 5 Pediatric Department, Buzzi Children’s Hospital, 20154 Milano, Italy; 6 Department of Biomedical and Clinical Science, University of Milano, 20157 Milano, Italy; 7 Department of Public Health, Experimental and Forensic Medicine, University of Pavia, Pavia, Italy; 8 Medical Direction, Fondazione IRCCS Policlinico San Matteo, Pavia, Italy; 9 Department of Internal Medicine and Therapeutics, University of Pavia, 27100 Pavia, Italy

**Keywords:** Pediatric obesity, Phenotype, Accelerometry, Insulin sensitivity, Cardiometabolic risk

## Abstract

Pediatric obesity is associated with early cardiometabolic risk, yet MVPA alone may not capture meaningful variation in movement behavior. We applied DDRTree to accelerometry data to identify multidimensional physical activity phenotypes in children and adolescents with obesity and assessed their associations with body composition and cardiometabolic. This cross-sectional study included 91 children and adolescents with obesity (43% girls), aged 6–17 years, who wore a thigh-mounted accelerometer for 7 days. Multidimensional accelerometry features were extracted and PA phenotypes identified using DDRTree, an unsupervised reversed graph-embedding method that maps high-dimensional data onto a low-dimensional branching manifold. DDRTree identified 3-PA phenotypes. Anthropometry, metabolic outcomes, and a continuous metabolic syndrome risk score were compared across phenotypes using ANCOVA adjusted for age and sex. DDRTree identified three PA phenotypes: Sedentary (24%), Light Activity (55%), and Higher Activity (21%); only three participants met MVPA recommendations. Compared with the Sedentary Pattern, the Higher Activity Pattern accumulated 122 min/day less sedentary time and 16.5 min/day more MVPA (*p* < 0.001) and showed lower fat mass percentage, higher fat-free mass percentage, higher insulin sensitivity (SPISE), and a lower metabolic risk score (*p* ≤ 0.05).

*Conclusion*: Multidimensional PA phenotypes derived from accelerometry identify clinically meaningful differences in body composition and metabolic risk beyond MVPA duration, supporting phenotype-based approaches for pediatric obesity risk stratification and intervention design.

**Supplementary Information:**

The online version contains supplementary material available at 10.1007/s00431-026-07299-2.

**Table:** 

**What is Known?** • *Most children and adolescents with obesity do not meet moderate-to-vigorous physical activity recommendations and are at increased risk of adverse cardiometabolic outcomes.* • *Physical activity volume alone may not fully capture health-relevant movement behaviour; sedentary accumulation, light activity, activity fragmentation, and transitions between activity states may also be associated with metabolic health.*
**What is New?** • *Multidimensional analysis of thigh-worn accelerometry data identified three distinct physical activity phenotypes, Sedentary, Light Activity, and Higher Activity, even though only three participants met recommended moderate-to-vigorous physical activity levels.* • *The Higher Activity phenotype was associated with lower fat mass percentage, higher fat-free mass percentage, greater insulin sensitivity, and a lower metabolic risk score than the Sedentary phenotype, supporting assessment beyond moderate-to-vigorous physical activity duration alone*.

## Background

Pediatric obesity is a major global public health concern, with prevalence rates continuing to rise worldwide and substantial implications for cardiometabolic health across the life course [1]. Children with obesity are at increased risk of developing insulin resistance, dyslipidemia, hypertension, and type 2 diabetes, often already detectable at a young age and likely to track into adulthood [2–5]. Identifying modifiable behavioral factors that contribute to metabolic risk in this population is therefore a critical research priority.

Physical activity (PA) is widely recognized as a cornerstone in the prevention and management of pediatric obesity and its metabolic complications. Current international guidelines recommend that children and adolescents accumulate at least 60 min per day of moderate-to-vigorous physical activity (MVPA) [6–8]. However, despite these recommendations, the vast majority of children with obesity fail to meet MVPA targets [7, 9, 10]. Indeed, an emerging body of evidence suggests that the health benefits of PA may not be fully captured by MVPA duration alone [11], specifically in populations characterized by overall low activity levels. Accelerometer-based studies have shown that sedentary behavior, light-intensity PA, and the temporal organization of movement throughout the day are independently associated with cardiometabolic health markers in children [12–14]. For example, prolonged uninterrupted sedentary time has been linked to insulin resistance and adverse lipid profiles, even after adjustment for MVPA [15, 16]. Similarly, higher fragmentation of sedentary time and more frequent transitions between activity states appear to be metabolically favorable [17, 18]. These findings challenge the traditional “minutes-based” paradigm and suggest that how PA is accumulated may be as important as how much is accumulated.

Advances in wearable technology and signal processing now allow the extraction of high-resolution accelerometry features that describe not only activity volume and intensity, but also movement variability, bout structure, inequality of activity distribution, and behavioral transitions across the day [19, 20]. However, integrating this multidimensional information into clinically meaningful constructs remains challenging.

Unsupervised machine learning and dimensionality reduction techniques offer a promising framework to address this limitation [21, 22]. In particular, discriminative dimensionality reduction via learning a tree (DDRTree) enables the projection of high-dimensional behavioral data into a low-dimensional manifold while preserving continuous trajectories and branching structures [23]. Unlike traditional clustering approaches that impose discrete group boundaries, DDRTree captures gradual transitions between behavioral states, making it particularly suitable for modeling PA as a continuum rather than a set of rigid categories [24, 25].

To date, few studies have applied such approaches to pediatric accelerometry data, and even fewer have examined whether data-driven PA phenotypes are associated with metabolic health beyond conventional MVPA metrics, especially in children with obesity [26]. Understanding whether distinct patterns of daily movement organization correspond to different metabolic risk profiles [24, 27] could have important implications for risk stratification and intervention design.

For these reasons, the aim of the present study was to identify distinct multidimensional PA phenotypes in children and adolescents with obesity using DDRTree applied to 7-day thigh-worn accelerometry data and to examine whether these phenotypes, beyond total MVPA duration, are associated with differences in body composition and cardiometabolic risk factors, independently of age and sex. We hypothesized that phenotypes characterized by lower sedentary accumulation and more dynamic movement patterns would exhibit a more favorable metabolic profile, thereby providing a potential advantage for early risk stratification and the development of more targeted and effective intervention strategies.

## Methods

### Study design

This study is a cross-sectional analysis conducted within the framework of the “Fight against Pediatric Obesity: from a predictive tool for type 2 Diabetes and Cardiovascular diseases risk to healthy educational programs” (PODiaCar) project. The study protocol was approved by the Ethics Committee Lombardia 1 (protocol number: CET 202–2023) and conducted in accordance with the Declaration of Helsinki [28].

Parents or legal guardians provided written informed consent before participation and verbal assent was obtained from all the children. Reporting of this cross-sectional analysis follows the Strengthening the Reporting of Observational Studies in Epidemiology (STROBE) guidelines (Supplementary Table S1).

### Participants

A total of 91 children and adolescents with obesity (39 girls, corresponding to 43% of the total sample) were included in the present analysis. Participants were enrolled within the PODiaCar project and completed both clinical assessments and PA monitoring. The recruitment process was executed at a pediatric outpatient clinic in Milan, Italy, following referrals from primary care pediatricians or general practitioners.

The inclusion criteria for this analysis were as follows: age between 6 and 17 years, obesity defined as a body mass index (BMI) *z*-score greater than 2 standard deviations according to World Health Organization reference standards, and availability of valid accelerometry data suitable for multidimensional feature extraction. Participants were required to be free from any medical conditions that could habitual PA participation. Exclusion criteria included secondary causes of obesity, chronic diseases affecting mobility or metabolism, recent musculoskeletal injuries, and incomplete anthropometric, biochemical, or accelerometry data. Participants with insufficient accelerometer wear time or poor-quality recordings were excluded from the final sample.

### Anthropometric characteristics

Anthropometric measurements included body weight, standing height, waist circumference, BMI, BMI *z*-score, waist-to-height ratio (WtHr), and estimated body composition. All measurements were performed by trained personnel following standardized procedures [29, 30]. Participants were assessed barefoot and wearing light clothing (shorts and a T-shirt). Body weight was measured to the nearest 0.1 kg using a calibrated electronic scale (Seca 864, Seca GmbH & Co., Hamburg, Germany). Standing height was measured to the nearest 0.1 cm using a wall-mounted stadiometer (Harpenden stadiometer, Holtain Ltd., Crymych, UK), with participants standing upright and head positioned in the Frankfurt plane. Waist circumference was measured using a non-elastic flexible tape, placed horizontally at the midpoint between the lowest rib margin and the iliac crest. BMI was calculated as body weight (kg) divided by height squared (m^2^). Age- and sex-specific BMI *z*-scores were derived using World Health Organization (WHO) growth reference standards. Obesity was defined as a BMI *z*-score greater than 2 standard deviations according to WHO criteria [31]. Body composition was estimated using validated anthropometric prediction equations developed by Hudda et al. [32], which estimate fat mass and fat-free mass based on age, sex, height, weight, and waist circumference. Fat mass percentage (FM%) and fat-free mass percentage (FFM%) were subsequently derived from these estimates.

### Clinical and biochemical assessments

Blood pressure and biochemical measurements were obtained under standardized conditions.

Resting blood pressure was measured in the morning after at least 5 min of seated rest using an appropriately sized cuff. Measurements were performed on the right arm with the participant in a seated position, back supported and feet flat on the floor. Systolic and diastolic blood pressure values were recorded as the average of two consecutive measurements taken at 3-min intervals [33]. Blood pressure *z*-scores were calculated using age-, sex-, and height-specific reference values [34].

Fasting venous blood samples were collected between 8:30 and 9:00 a.m. after an overnight fast to assess metabolic parameters. Samples were analyzed on the same day using standardized automated methods (Advia XPT system, Siemens Healthcare Diagnostics). Biochemical measurements included fasting plasma glucose, total cholesterol, high-density lipoprotein cholesterol (HDL-C), low-density lipoprotein cholesterol (LDL-C), triglycerides, and fasting insulin concentrations.

Insulin resistance was estimated using surrogate indices such as homeostatic model assessment for insulin resistance (HOMA-IR), calculated as fasting insulin (µU/mL) multiplied by fasting glucose (mg/dL) divided by 22.5 [35]; the triglyceride–glucose (TyG) index, calculated as ln [fasting triglycerides (mg/dL) × fasting glucose (mg/dL)/2] and its derivative, the TyG-zBMI index, calculated as TyG × zBMI, were also assessed [36]. Insulin sensitivity was additionally assessed using the single-point insulin sensitivity estimator (SPISE), computed according to the equation proposed by Paulmichl et al. [37] and by Barchetta et al. for the Italian population [38]:

$$SPISE=600\times HDL-{C}^{0.185}/\left(triglyceride{s}^{0.2}\times BM{I}^{1.338}\right)$$

where HDL-C and triglycerides are expressed in mg/dL and BMI in kg/m^2^. Higher values represented better insulin sensibility.

To evaluate overall cardiometabolic risk, a continuous metabolic risk score (MetS *z*-score) was computed based on sex-specific equations incorporating BMI *z*-score, HDL-C, systolic blood pressure, triglycerides (log-transformed), and fasting glucose [39]. The equations for the MetS *z*-score are as follows:

For boys:

$$\mathrm{M}\mathrm{e}\mathrm{t}\mathrm{S} \mathrm{r}\mathrm{i}\mathrm{s}\mathrm{k} \mathrm{s}\mathrm{c}\mathrm{o}\mathrm{r}\mathrm{e}\hspace{0.17em}=\hspace{0.17em}\hspace{0.17em}-\hspace{0.17em}4.931\hspace{0.17em}+\hspace{0.17em}0.2804 * \mathrm{B}\mathrm{M}\mathrm{I} z-\mathrm{s}\mathrm{c}\mathrm{o}\mathrm{r}\mathrm{e}\hspace{0.17em}-\hspace{0.17em}0.0257 * \mathrm{H}\mathrm{D}\mathrm{L}-\mathrm{C}\hspace{0.17em}+\hspace{0.17em}0.0189 * \mathrm{S}\mathrm{y}\mathrm{s}\mathrm{t}\mathrm{o}\mathrm{l}\mathrm{i}\mathrm{c} \mathrm{b}\mathrm{l}\mathrm{o}\mathrm{o}\mathrm{d} \mathrm{p}\mathrm{r}\mathrm{e}\mathrm{s}\mathrm{s}\mathrm{u}\mathrm{r}\mathrm{e}\hspace{0.17em}+\hspace{0.17em}0.6240 * \mathrm{l}\mathrm{o}\mathrm{g}(\mathrm{t}\mathrm{r}\mathrm{i}\mathrm{g}\mathrm{l}\mathrm{y}\mathrm{c}\mathrm{e}\mathrm{r}\mathrm{i}\mathrm{d}\mathrm{e}\mathrm{s})\hspace{0.17em}+\hspace{0.17em}0.0140 * \mathrm{f}\mathrm{a}\mathrm{s}\mathrm{t}\mathrm{i}\mathrm{n}\mathrm{g} \mathrm{g}\mathrm{l}\mathrm{u}\mathrm{c}\mathrm{o}\mathrm{s}\mathrm{e}$$

For girls:

$$\mathrm{M}\mathrm{e}\mathrm{t}\mathrm{S} \mathrm{r}\mathrm{i}\mathrm{s}\mathrm{k} \mathrm{s}\mathrm{c}\mathrm{o}\mathrm{r}\mathrm{e}\hspace{0.17em}=\hspace{0.17em}\hspace{0.17em}-\hspace{0.17em}4.3757\hspace{0.17em}+\hspace{0.17em}0.4849 * \mathrm{B}\mathrm{M}\mathrm{I} z-\mathrm{s}\mathrm{c}\mathrm{o}\mathrm{r}\mathrm{e}\hspace{0.17em}-\hspace{0.17em}0.0176 * \mathrm{H}\mathrm{D}\mathrm{L}-\mathrm{C}\hspace{0.17em}+\hspace{0.17em}0.0257 * \mathrm{S}\mathrm{y}\mathrm{s}\mathrm{t}\mathrm{o}\mathrm{l}\mathrm{i}\mathrm{c} \mathrm{b}\mathrm{l}\mathrm{o}\mathrm{o}\mathrm{d} \mathrm{p}\mathrm{r}\mathrm{e}\mathrm{s}\mathrm{s}\mathrm{u}\mathrm{r}\mathrm{e}\hspace{0.17em}+\hspace{0.17em}0.3172 * \mathrm{l}\mathrm{o}\mathrm{g}(\mathrm{t}\mathrm{r}\mathrm{i}\mathrm{g}\mathrm{l}\mathrm{y}\mathrm{c}\mathrm{e}\mathrm{r}\mathrm{i}\mathrm{d}\mathrm{e}\mathrm{s})\hspace{0.17em}+\hspace{0.17em}0.0083 * \mathrm{f}\mathrm{a}\mathrm{s}\mathrm{t}\mathrm{i}\mathrm{n}\mathrm{g} \mathrm{g}\mathrm{l}\mathrm{u}\mathrm{c}\mathrm{o}\mathrm{s}\mathrm{e}$$

Higher values of the MetS *z*-score reflect a greater metabolic risk.

### Physical activity assessment

PA and sedentary behavior were objectively assessed using a triaxial thigh-mounted accelerometer (Fibion®, Fibion Inc., Jyväskylä, Finland) [40]. Participants wore the device on the thigh for seven consecutive days. The device’s placement and utilization were meticulously demonstrated by trained research personnel, and written instructions were provided to accompany a video explanation. Raw acceleration data were processed using the open-source GGIR package in R following standardized procedures [40]. Signals were auto-calibrated to local gravity, aggregated into 5-s epochs, and summarized using the Euclidean Norm Minus One (ENMO, mg), with negative values truncated to zero. Non-wear periods were identified based on low signal variability and excluded from analyses.

Time spent in sedentary, light, moderate, and vigorous activity, as well as total moderate-to-vigorous PA (MVPA), was derived from ENMO during waking hours. In addition, all temporal organization and fragmentation metrics, including activity bout duration, sedentary accumulation, activity transitions, and inequality indices (e.g., Gini coefficients), were computed directly from raw accelerometry using GGIR-based algorithms [41]. Weighted daily summaries of activity volume, intensity, and temporal structure were subsequently used for multidimensional feature extraction and DDRTree-based behavioral phenotype identification.

### Statistical analysis

Continuous variables are summarized as estimated marginal means or raw means with 95% confidence intervals (CI), as appropriate. Differences in accelerometry-derived behavioral metrics, anthropometric characteristics, body composition, and metabolic outcomes across PA phenotypes were evaluated using analysis of covariance (ANCOVA), with phenotype as the main fixed factor and age and sex included as covariates. Pairwise comparisons between phenotypes were conducted using Tukey-adjusted post hoc tests.

Accelerometry-derived behavioral features obtained from GGIR, including activity volume, intensity distribution, sedentary accumulation, temporal fragmentation indices, and activity transition metrics, were standardized prior to dimensionality reduction. Behavioral phenotypes were identified using discriminative dimensionality reduction via learning a tree (DDRTree), an unsupervised reversed graph-embedding approach that projects high-dimensional behavioral data into a low-dimensional manifold while preserving continuous trajectories and branching structure. The optimal number of phenotypes was determined using silhouette analysis, while cluster robustness was evaluated through repeated subsampling using the Adjusted Rand Index (ARI).

Adjusted comparisons of anthropometric, body composition, and metabolic outcomes across phenotypes are reported as estimated marginal means and mean differences with 95% confidence intervals derived from ANCOVA models, adjusting for age and sex. Graphical representations display observed raw data distributions alongside adjusted group-level comparisons.

To assess the robustness of primary findings, Bayesian sensitivity analyses were performed for anthropometric and metabolic outcomes. Bayesian Gaussian regression models with weakly informative priors were fitted, including phenotype as the primary predictor and adjusting for age and sex. Posterior adjusted means and 95% credible intervals were estimated for each phenotype, along with posterior mean differences between groups (Supplementary Materials).

All statistical tests were two-sided, with significance set at *p* < 0.05. Analyses were conducted using R software (version 4.5.1; R Foundation for Statistical Computing, Vienna, Austria). Accelerometry data processing was performed using the *GGIR* package (version 3.3–1). Dimensionality reduction and phenotype identification were implemented using the DDRTree algorithm within the *monocle* package (version 2.38.0). Estimated marginal means and pairwise contrasts were obtained using the *emmeans* package (version 2.0.0). Bayesian sensitivity analyses were conducted using the *arm* package, and data visualization was performed using *ggplot2* (version 4.0.1).

## Results

Descriptive characteristics of the children included in the study are presented in Table 1.

**Table 1:** Descriptive characteristics of the total sample, divided by sex

	Girls (*n* = 39)	Boys (*n* = 52)	All (*n* = 91)
Age (years)	11.0 (10.1, 11.8)	11.3 (10.7, 11.9)	11.2 (10.7, 11.6)
Height (cm)	148.7 (144.5, 152.9)	151.2 (147.7, 154.6)	150.1 (147.5, 152.7)
Weight (kg)	66.1 (59.8, 72.5)	66.3 (61.3, 71.3)	66.2 (62.3, 70.1)
BMI *z*-score	2.9 (2.8, 3.1)	3.1 (2.9, 3.3)	3.1 (2.9, 3.2)
Waist circumference (cm)	85.8 (81.6, 90.0)	89.1 (86.0, 92.2)	87.7 (85.2, 90.2)
WtHr	0.6 (0.6, 0.6)	0.6 (0.6, 0.6)	0.6 (0.6, 0.6)
Fat mass (kg)	28.6 (25.4, 31.8)	26.0 (23.8, 28.2)	27.1 (25.3, 28.9)
Fat mass (%)	42.7 (41.5, 44.0)	39.0 (38.0, 40.0)	40.6 (39.7, 41.5)
Fat free mass (%)	57.3 (56.0, 58.5)	61.0 (60.0, 62.0)	59.4 (58.5, 60.3)
Systolic blood pressure (mmHg)	109.2 (105.8, 112.5)	109.0 (106.6, 111.3)	109.0 (107.1, 111.0)
Diastolic blood pressure (mmHg)	70.1 (67.5, 72.7)	68.5 (66.4, 70.6)	69.2 (67.6, 70.8)
Fasting glycemia (mg/dL)	91.9 (88.0, 95.7)	89.0 (87.4, 90.6)	90.2 (88.4, 92.1)
Fasting insulin (U/mL)	19.0 (15.9, 22.1)	19.2 (17.2, 21.2)	19.1 (17.4, 20.8)
HOMA-IR	4.4 (3.7, 5.1)	4.2 (3.8, 4.7)	4.3 (3.9, 4.7)
SPISE	5.7 (5.3, 6.1)	5.7 (5.4, 6.1)	5.7 (5.4, 5.9)
Triglycerides (mg/dL)	102.5 (81.6, 123.3)	95.5 (87.5, 103.5)	98.5 (88.6, 108.3)
LDL-C (mg/dL)	80.9 (70.2, 91.7)	83.6 (77.9, 89.3)	82.4 (76.9, 88.0)
HDL-C (mg/dL)	47.2 (43.7, 50.8)	43.2 (40.5, 45.8)	44.9 (42.8, 47.1)
Total cholesterol (mg/dL)	156.2 (148.2, 164.2)	143.5 (135.8, 151.2)	149.0 (143.3, 154.6)
TyG index	8.3 (8.2, 8.5)	8.3 (8.2, 8.4)	8.3 (8.2, 8.4)
TyG-zBMI index	24.4 (22.9, 26.0)	26.1 (24.3, 27.8)	25.4 (24.2, 26.6)
MetS *z*-score	1.2 (1.0, 1.4)	1.0 (0.8, 1.1)	1.1 (1.0, 1.2)

All data are presented as mean (95% confidence interval, CI). *BMI*, body mass index; *WtHr*, waist-to-height-ratio; *HOMA-IR*, homeostatic model assessment for insulin resistance; *SPISE*, single-point insulin sensibility estimator; *LDL-C*, low-density lipoprotein; *HDL-C*, high-density lipoprotein.

**Fig. 1:**
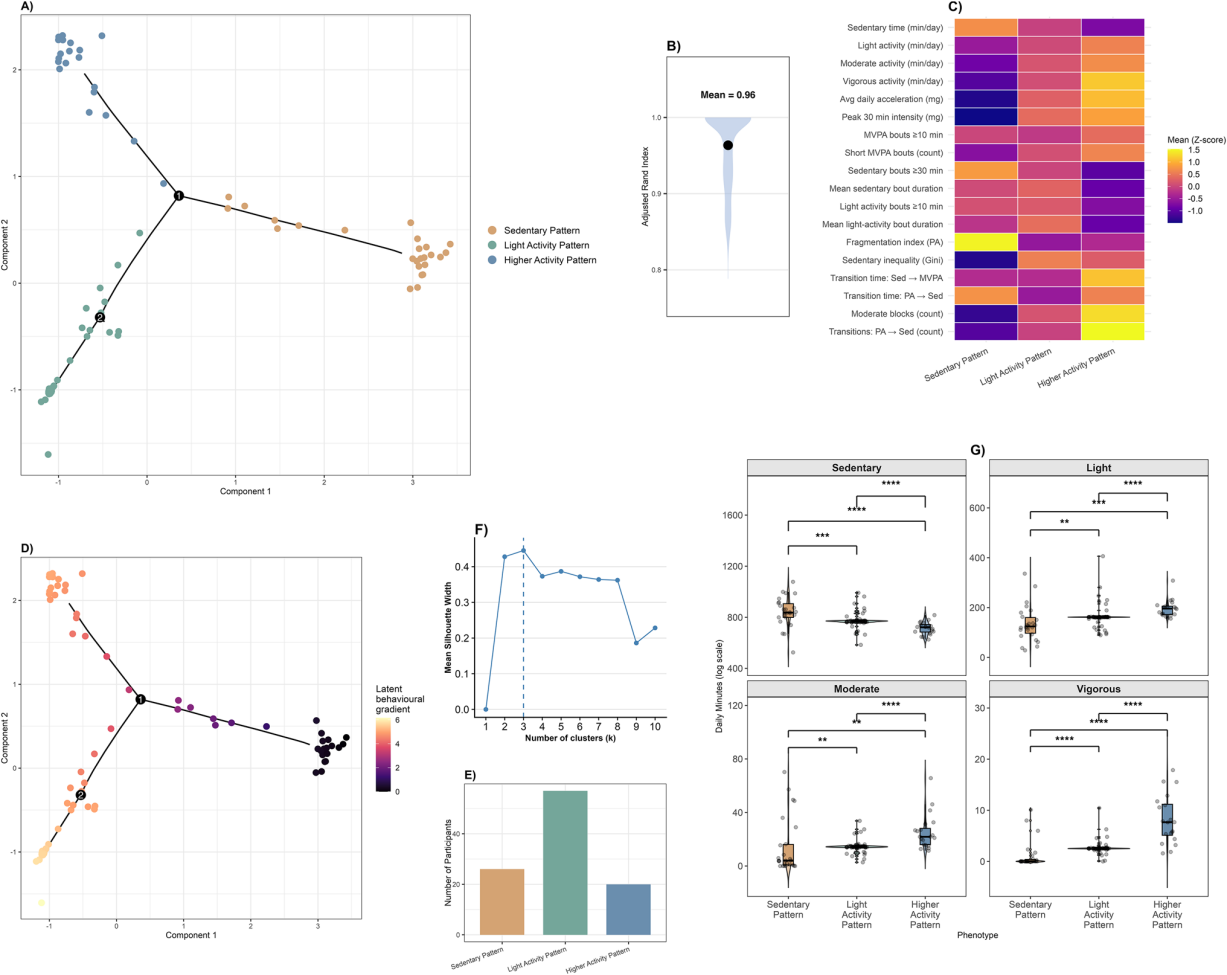
Multidimensional accelerometry features and DDRTree-derived behavioral phenotypes in children with obesity. **A** DDRTree manifold showing three latent behavioral phenotypes, Sedentary Pattern, Light Activity Pattern, and Higher Activity Pattern, identified from 7-day accelerometry. Points represent individual participants, and branch topology reflects major gradients in activity behavior. **B** Violin plot of the Adjusted Rand Index (ARI). The central black point marks the mean ARI. **C** Heat map of accelerometry-derived features (intensity distribution, sedentary fragmentation, bout structure, transitions, and inequality metrics) illustrating distinct multivariate activity signatures associated with each phenotype. **D** DDRTree manifold colored by latent component values, highlighting behavioral gradients captured by the embedding. **E** Number of children with obesity for each phenotype. **F** Silhouette analysis supporting an optimal clustering solution of *k* = 3. **G** Group differences in daily time spent in sedentary, light, moderate, and vigorous activity, adjusted for age and sex. Boxes show median and interquartile range (IQR); whiskers denote 1.5 × IQR. *p* values reflect ANOVA with Tukey post hoc tests

Figure 1 represents the model used to estimate the phenotypes of PA. In our sample of children with obesity, DDRTree applied to 7-day accelerometry-derived features generated a structured low-dimensional manifold revealing three distinct behavioral phenotypes (*k* = 3; Fig. 1A). Participants were distributed along branching trajectories reflecting gradients in daily movement behavior and were classified as Sedentary Pattern, Light Activity Pattern, or Higher Activity Pattern. Cluster stability across resampling was high, as indicated by a consistently elevated Adjusted Rand Index (ARI, Fig. 1B), and silhouette analysis confirmed that a three-cluster solution maximized within-cluster similarity and between-cluster separation (Fig. 1F).

The three phenotypes displayed clearly distinct multivariate accelerometry profiles (Fig. 1C). Compared with the Sedentary Pattern, the Higher Activity Pattern showed lower sedentary accumulation, greater activity fragmentation, more frequent intensity transitions, and a more even distribution of activity across intensities, whereas the Light Activity Pattern exhibited intermediate values across most feature domains. Visualization of latent component values on the DDRTree embedding revealed smooth transitions along the manifold, suggesting that behavioral variation is organized along continuous trajectories rather than abrupt category boundaries (Fig. 1D).

Phenotype sizes were unequal (Fig. 1E), with most children (55%) classified into the Light Activity Pattern, and a smaller subgroup assigned to the Higher Activity Pattern (21%) and to the Sedentary Pattern (24%). Notably, only 3 participants met WHO-recommended MVPA levels, yet significant differences in daily activity composition emerged across phenotypes after adjustment for age and sex (Fig. 1G). Compared with the Sedentary Pattern, children in the Higher Activity Pattern accumulated approximately 122 min/day less sedentary time (*p* < 0.0001) and 16.5 min/day more MVPA (*p* < 0.001), alongside significantly higher time spent in light and moderate activity (all *p* < 0.001). The Light Activity Pattern consistently showed intermediate activity levels.

**Fig. 2:**
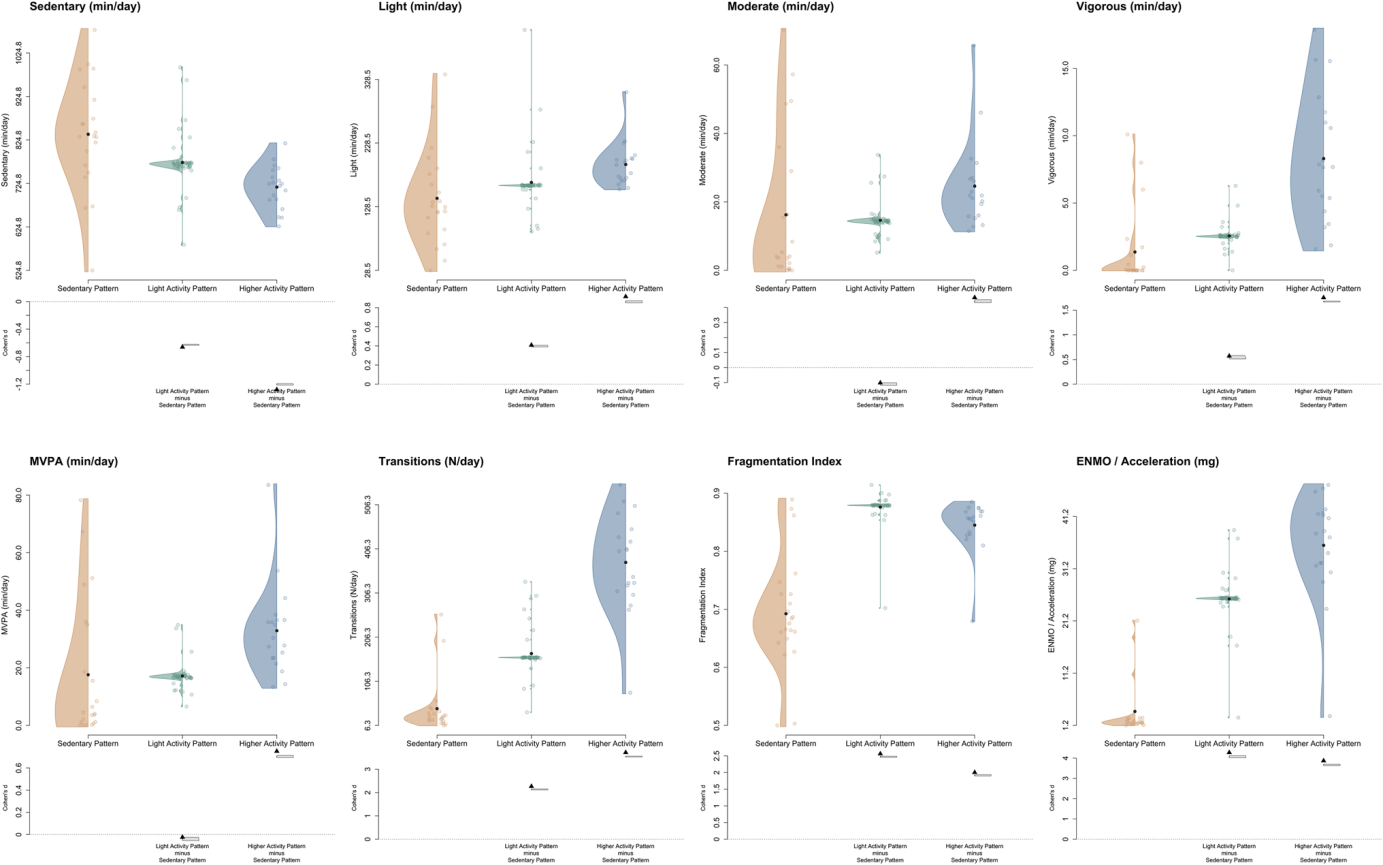
Behavioral characteristics of physical activity phenotypes. Violin plots illustrate the distribution of accelerometer-derived behavioral metrics across the three DDRTree-derived phenotypes (Sedentary Pattern, Light Activity Pattern, and Higher Activity Pattern). Panels show daily time spent in sedentary, light, moderate, and vigorous physical activity; total moderate to vigorous activity (MVPA); number of daily activity transitions; temporal fragmentation (GINI index); and overall activity intensity (ENMO, mg). Individual data points are overlaid, with boxplots indicating median and interquartile range

Marked differences in accelerometry-derived behavioral characteristics were observed across the three DDRTree-derived phenotypes (Fig. 2). Daily sedentary time progressively decreased from the Sedentary Pattern to the Light Activity Pattern and was lowest in the Higher Activity Pattern, whereas time spent in light, moderate, and vigorous PA showed the opposite trend. Children classified in the Higher Activity Pattern accumulated substantially more moderate and vigorous activity and less sedentary time compared with the Sedentary Pattern, with the Light Activity Pattern consistently occupying an intermediate position. Similar gradients were evident for total MVPA and overall activity intensity (ENMO), indicating that higher phenotype classification was associated with both greater activity volume and intensity.

Beyond volume-based metrics, phenotypes differed markedly in movement organization. The number of daily activity transitions increased across phenotypes, with the Higher Activity Pattern exhibiting the greatest frequency of transitions, suggesting more dynamic and frequently changing activity behavior. Temporal fragmentation, quantified by the Gini index, also differed significantly between groups, with lower inequality in activity accumulation observed in more active phenotypes. Notably, several of these differences were already apparent between the Sedentary and Light Activity Patterns, despite relatively modest differences in MVPA, highlighting the contribution of temporal structure and intensity distribution to phenotype differentiation.

Consistent with these observations, Supplementary Figs. S1 and S2 show that phenotype separation was driven by a combination of intensity-, volume-, and temporal structure-related metrics. Metrics related to higher-intensity activity, overall acceleration (ENMO), and activity transitions displayed the largest effect sizes (partial *η*^2^), frequently exceeding thresholds for large effects, whereas traditional MVPA-duration measures accounted for a smaller proportion of between-phenotype variance.

**Fig. 3:**
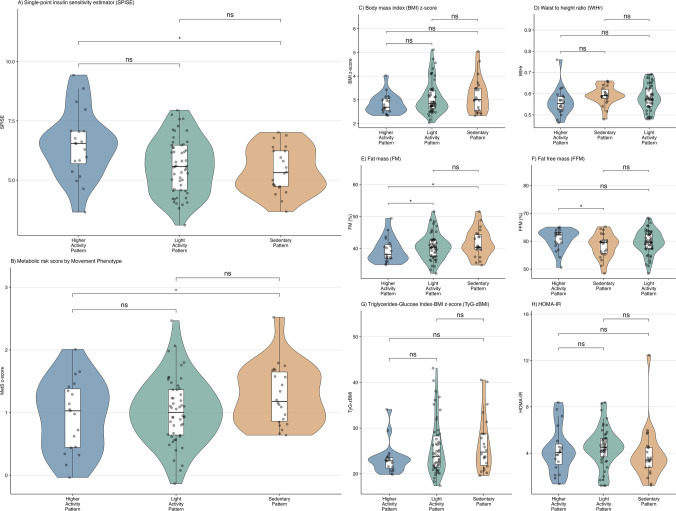
Metabolic characteristics across DDRTree-derived activity phenotypes. Panels show differences in single-point insulin sensitivity estimator (SPISE, **A**), metabolic risk score (MetS risk score, **B**), body mass index *z*-score (BMI *z*-score, **C**), waist-to-height ratio (WtHr, **D**), fat mass percentage (FM%, **E**), fat-free mass percentage (FFM%, **F**), triglyceride–glucose index BMI *z*-score (TyG-zBMI, **G**), and homeostatic model assessment for insulin resistance (HOMA-IR, **H**) between the three activity phenotypes. Violin plots display the distribution of observed values, overlaid with boxplots indicating the median and interquartile range. Horizontal brackets denote pairwise comparisons. Two-sided *p* values were obtained from analysis of covariance (ANCOVA, parametric or non-parametric depending on variable distribution) adjusted for age and sex

Adjusted comparisons of anthropometric, body composition, and metabolic outcomes across DDRTree-derived PA phenotypes are summarized in Table 2 and visualized in Fig. 3. Overall, children classified within the Higher Activity Pattern exhibited a better body composition and metabolic profile compared with both Light Activity and Sedentary patterns, while anthropometric measures did not differ significantly across groups.

**Table 2:** Differences in BMI *z*-score, body composition, blood pressure, glucose homeostasis, insulin sensitivity, and blood lipid profile in the three phenotypes

	High activity pattern	Light activity pattern	Sedentary pattern	High activity vs sedentary		High activity vs light activity		Sedentary vs light activity	
	Mean (95% CI)	Mean (95% CI)	Mean (95% CI)	Mean difference (95% CI)	*p* values	Mean difference (95% CI)	*p* values	Mean difference (95% CI)	*p* values
Height (cm)	151.3 (147.7, 155.0)	150.3 (148.0, 152.6)	148.2 (144.9, 151.5)	3.2 (− 2.7, 9.1)	0.401	1.0 (− 4.3, 6.3)	0.895	2.2 (− 2.7, 7.0)	0.54
Weight (kg)	65.01 (59.8, 70.3)	67.5 (64.1, 70.8)	65.1 (60.3, 69.8)	− 0.0 (− 8.5, 8.4)	1.000	− 2.4 (− 10.1, 5.3)	0.736	2.4 (− 4.6, 9.4)	0.698
BMI ***z***-score	2.75 (2.46, 3.04)	3.11 (2.93, 3.29)	3.14 (2.88, 3.41)	− 0.39 (− 0.86, 0.07)	0.115	− 0.36 (− 0.78, 0.07)	0.115	− 0.04 (− 0.42, 0.35)	0.974
Waist circumference (cm)	86.0 (81.3, 90.7)	88.1 (85.1, 91.0)	87.7 (83.5, 92.0)	− 1.7 (− 9.2, 5.9)	0.854	− 2.0 (− 8.9, 4.9)	0.764	0.3 (− 5.9, 6.6)	0.992
WtHr	0.57 (0.54, 0.59)	0.58 (0.57, 0.60)	0.59 (0.57, 0.62)	− 0.03 (− 0.07, 0.02)	0.289	− 0.02 (− 0.06, 0.02)	0.533	− 0.01 (− 0.05, 0.03)	0.800
Fat mass (kg)	25.5 (22.5, 28.5)	28.2 (26.3, 30.1)	27.1 (24.4, 29.9)	− 1.6 (− 6.5, 3.3)	0.711	− 2.7 (− 7.1, 1.7)	0.314	1.1 (− 2.9, 5.1)	0.790
Fat mass (%)	38.79 (37.08, 40.49)	41.40 (40.33, 42.47)	41.57 (40.03, 43.10)	− 2.78 (− 5.51, − 0.05)	**0.045**	− 2.62 (− 5.10, − 0.13)	0.037	− 0.16 (− 2.43, 2.10)	0.984
Fat free mass (%)	61.21 (59.51, 62.92)	58.60 (57.53, 59.67)	58.43 (56.90, 59.97)	2.78 (0.05, 5.51)	**0.045**	2.62 (0.13, 5.10)	0.037	0.16 (− 2.10, 2.43)	0.984
Systolic blood pressure (***z***-score)	− 0.03 (− 0.44, 0.38)	0.58 (0.32, 0.83)	0.45 (0.08, 0.82)	− 0.48 (− 1.14, 0.18)	0.193	− 0.60 (− 1.20, − 0.01)	**0.048**	0.12 (− 0.42, 0.67)	0.856
Diastolic blood pressure (***z***-score)	0.49 (0.16, 0.81)	0.72 (0.51, 0.93)	0.77 (0.47, 1.06)	− 0.28 (− 0.80, 0.24)	0.415	− 0.23 (− 0.71, 0.25)	0.480	− 0.05 (− 0.48, 0.39)	0.963
Fasting glycemia (mg/dL)	92 (88, 96)	91 (88, 94)	88 (89, 92)	3 (− 3, 10)	0.430	1 (− 5, 7)	0.953	3 (− 3, 8)	0.469
Fasting insulin (U/mL)	17.88 (13.98, 21.78)	20.15 (17.69, 22.60)	17.97 (14.45, 21.49)	− 0.09 (− 6.34, 6.17)	0.999	− 2.27 (− 7.96, 3.42)	0.610	2.18 (− 3.00, 7.36)	0.576
HOMA-IR	4.1 (3.2, 5.0)	4.6 (4.0, 5.2)	3.9 (3.1, 4.7)	0.24 (− 1.21, 1.68)	0.920	− 0.45 (− 1.77, 0.86)	0.691	0.69 (− 0.51, 1.89)	0.360
SPISE	6.2 (5.8, 6.7)	5.6 (5.3, 5.9)	5.3 (4.9, 5.8)	0.9 (0.1, 1.7)	**0.016**	0.6 (− 0.1, 1.3)	0.080	0.3 (− 0.4, 0.9)	0.577
Triglycerides (mg/dL)	96 (74, 118)	95 (81, 109)	111 (91, 131)	− 15 (− 51, 21)	0.592	1 (− 31, 34)	0.994	− 16 (− 46, 14)	0.399
LDL-C (mg/dL)	73 (61, 86)	86 (78, 94)	82 (71, 93)	− 9 (− 29, 11)	0.552	− 13 (− 31, 5)	0.209	4 (− 12, 21)	0.813
HDL-C (mg/dL)	46 (41, 50)	47 (44, 49)	42 (38, 46)	4 (− 4, 11)	0.435	− 1 (− 7, 6)	0.955	5 (− 1, 11)	0.172
Total cholesterol (mg/dL)	142 (130, 155)	153 (145, 161)	149 (138, 161)	− 7 (− 27, 13)	0.659	− 11 (− 29, 7)	0.317	4 (− 13, 20)	0.848
TyG index	8.22 (8.02, 8.42)	8.31 (8.18, 8.44)	8.38 (8.20, 8.56)	− 0.16 (− 0.48, 0.17)	0.479	− 0.09 (− 0.38, 0.21)	0.752	− 0.07 (− 0.34, 0.20)	0.814
TyG-zBMI index	22.5 (20.0, 25.0)	25.9 (24.3, 27.4)	26.3 (24.1, 28.6)	− 3.80 (− 7.78, 0.17)	0.064	− 3.4 (− 7.0, 0.3)	0.075	− 0.5 (− 3.8, 2.8)	0.942
MetS ***z***-score	0.82 (0.60, 1.04)	1.12 (0.98, 1.26)	1.24 (1.04, 1.45)	− 0.42 (− 0.78, − 0.06)	**0.017**	− 0.30 (− 0.63, 0.03)	0.080	− 0.12 (− 0.42, 0.17)	0.583

Data are presented as estimated marginal means and 95% confidence interval (CI) for descriptive values, and as estimated marginal mean differences with 95% CIs between groups. Two-sided *p* values are derived from analysis of covariance evaluating the differences between groups after adjusting for age and sex. *BMI*, body mass index; *WtHr*, waist-to-height-ratio; *HOMA-IR*, homeostatic model assessment for insulin resistance; *SPISE*, single-point insulin sensibility estimator; *LDL-C*, low-density lipoprotein; *HDL-C*, high-density lipoprotein

The boldface entries in Table 2 indicate statistically signifi cant results (*p* 0.05)

Although BMI *z*-score and WtHr did not reach statistical significance, both measures showed a graded pattern across phenotypes, with progressively lower values observed in the Higher Activity Pattern (BMI *z*-score: 2.75 [95% CI 2.46, 3.04]) compared with the Light (3.11 [2.93, 3.29]) and Sedentary patterns (3.14 [2.88, 3.41]) (Fig. 3C and D).

In contrast, significant differences were observed for body composition, with the Higher Activity Pattern showing a lower fat mass percentage (38.8%) and a higher fat-free mass percentage (61.2%) compared with both Light and Sedentary patterns (*p* = 0.045 and *p* = 0.037, respectively; Table 2; Fig. 3E and F).

Considering metabolic indices, insulin sensitivity estimated by SPISE was significantly higher in the Higher Activity Pattern compared with the Sedentary Pattern (mean difference = 0.90 [95% CI 0.14, 1.66], *p* = 0.016), with intermediate values observed in the Light Activity Pattern (Fig. 3A). Similarly, the composite metabolic risk score (MetS *z*-score) was significantly lower in the Higher Activity Pattern compared with the Sedentary Pattern (mean difference =  − 0.42 [− 0.78 to − 0.06], *p* = 0.017), indicating a lower overall cardiometabolic risk burden (Fig. 3B).

No statistically significant differences were detected across phenotypes for fasting glucose, insulin, HOMA-IR, blood pressure, or lipid concentrations, although most markers showed directionally consistent trends toward a more favorable profile in the Higher Activity Pattern (Table 2; Fig. 3G and H).

Bayesian sensitivity analyses using weakly informative priors yielded estimates that were directionally consistent with frequentist models for adiposity and metabolic outcomes (Supplementary Table S2). Indeed, posterior estimates supported lower fat mass percentage, lower TyG-zBMI index, higher SPISE values, and lower MetS *z*-scores in the Higher Activity Pattern compared with both Light and Sedentary patterns, while highlighting substantial uncertainty for individual cardiometabolic biomarkers.

## Discussion

In this study, we identified distinct multidimensional PA phenotypes in children and adolescents with obesity using a data-driven approach applied to high-resolution accelerometry data and demonstrated that multidimensional movement profiles captured heterogeneity in metabolic health that was not reflected by MVPA guideline attainment alone. These findings align with and extend a growing body of literature questioning the adequacy of MVPA alone as a marker of health-related PA behavior in pediatric populations [26, 42, 43]. Personalized approaches based on behavioral phenotypes could improve the early identification of individuals at higher metabolic risk and guide more targeted and effective interventions.

Previous studies have consistently shown that most children with obesity do not achieve recommended MVPA levels, limiting the discriminatory power of guideline-based thresholds in this population [7, 44]. Our results support this evidence, as only a very small fraction of participants met WHO recommendations, yet substantial heterogeneity in metabolic risk was still observed. This supports earlier observations that health-relevant behavioral variation persists even below conventional MVPA cut-points and may be captured by alternative dimensions of movement behavior.

The observed associations between more dynamic activity patterns and improved metabolic profiles are consistent with prior evidence linking sedentary behavior and its accumulation to cardiometabolic risk independently of MVPA [15]. Large observational studies have reported that prolonged uninterrupted sedentary time is associated with higher insulin resistance and adverse lipid profiles in children and adolescents, even after adjustment for MVPA [45]. Similarly, greater fragmentation of sedentary time and more frequent activity breaks have been associated with improved glucose metabolism and adiposity markers [46]. Our findings extend these results by showing that such features do not act in isolation but cluster into coherent behavioral phenotypes that collectively relate to metabolic health.

Importantly, differences between phenotypes were not driven only by activity volume but by a combination of reduced sedentary accumulation, higher frequency of transitions, and a more even distribution of activity intensity across the day. This observation aligns with recent accelerometry studies emphasizing the relevance of movement variability and intensity distribution, rather than sustained high-intensity activity alone, for metabolic regulation [47, 48]. Accordingly, our results indicate more favorable insulin sensitivity profiles in children characterized by higher overall activity levels and greater activity fragmentation, suggesting beneficial metabolic effects of distributed PA patterns. Mechanistically, frequent low- to moderate-intensity muscle contractions may repeatedly stimulate glucose uptake and lipid oxidation, thereby improving insulin sensitivity even in the absence of prolonged MVPA bouts [49].

The lack of significant differences in BMI *z*-score across phenotypes, despite clear contrasts in fat mass percentage and metabolic risk, is also consistent with previous literature highlighting the limitations of BMI as a marker of metabolic health in children [50, 51]. Several studies have shown that BMI poorly reflects changes in body composition and cardiometabolic risk, particularly during growth and puberty [52, 53]. Our findings reinforce the notion that behavior-related metabolic benefits may occur independently of measurable changes in BMI and underscore the value of incorporating body composition and metabolic indices into pediatric obesity research.

From a methodological perspective, our use of DDRTree adds to a small but emerging literature applying unsupervised machine learning techniques to PA data. While clustering approaches such as *k*-means or latent class analysis have previously been used to identify activity patterns in youth [54, 55], these methods typically impose discrete class boundaries. In contrast, the continuous trajectories observed in our DDRTree embedding are conceptually consistent with recent work in behavioral epidemiology suggesting that PA behaviors exist along continua rather than in mutually exclusive categories [56]. This continuous representation may better reflect real-world behavior and offers a flexible framework for capturing gradual behavioral change [22, 24].

The clinical and public health implications of these findings are in line with recent calls to broaden PA recommendations beyond MVPA-centric messages. Several authors have argued that encouraging children to “sit less and move more, more often” [57] may be a more achievable and inclusive strategy, particularly for those with obesity or low fitness levels [58]. Our results provide empirical support for this perspective by showing that children characterized by more dynamic, less sedentary activity patterns exhibit a more favorable metabolic profile, even without substantial differences in MVPA.

Some limitations should be interpreted in the context of the existing literature. The cross-sectional design prevents causal inference, as also noted in most observational accelerometry studies in pediatric populations. Additionally, while the sample size is comparable to or larger than several accelerometry-based phenotype studies, it limits exploration of sex- and age-specific trajectories, which have been shown to influence both activity behavior and metabolic risk [59]. Another limitation is that biological maturation was not assessed in the present study. Because maturational status may influence both physical activity participation and cardiometabolic risk independently of chronological age [60–62], residual confounding related to pubertal development cannot be excluded. Future studies with larger samples should incorporate measures of biological maturation, such as pubertal stage, maturity offset, or bio-banding approaches, to examine whether maturation moderates or mediates the associations between physical activity phenotypes and metabolic health. Finally, although body composition was estimated using validated anthropometric equations, direct measures such as dual-energy X-ray absorptiometry could provide more precise estimates in future studies.

## Conclusions

In agreement with and extending prior literature, this study demonstrates that multidimensional, data-driven PA phenotypes capture meaningful behavioral variation associated with metabolic health in children with obesity, beyond traditional MVPA metrics. These findings support a shift toward a more nuanced conceptualization of PA behavior that integrates sedentary patterns, movement dynamics, and intensity distribution, and they highlight the potential value of phenotype-based approaches for risk stratification and intervention development in pediatric obesity.

Supplementary information.

## Supplementary Information

Below is the link to the electronic supplementary material.

Supplementary file1 (DOCX 491 KB)

## Data Availability

The datasets used and/or analysed during the current study are available from the corresponding author on reasonable request.
